# Driving Competence in Mild Dementia with Lewy Bodies: In Search of Cognitive Predictors Using Driving Simulation

**DOI:** 10.1155/2015/806024

**Published:** 2015-12-02

**Authors:** Stephanie Yamin, Arne Stinchcombe, Sylvain Gagnon

**Affiliations:** ^1^School of Psychology, University of Ottawa, Canada; ^2^Faculty of Human Sciences, Saint Paul University, Room G-321, 223 Main Street, Ottawa, ON, Canada K1S 1C4; ^3^Centre for Research on Safe Driving, Lakehead University, Canada

## Abstract

Driving is a multifactorial behaviour drawing on multiple cognitive, sensory, and physical systems. Dementia is a progressive and degenerative neurological condition that impacts the cognitive processes necessary for safe driving. While a number of studies have examined driving among individuals with Alzheimer's disease, less is known about the impact of Dementia with Lewy Bodies (DLB) on driving safety. The present study compared simulated driving performance of 15 older drivers with mild DLB with that of 21 neurologically healthy control drivers. DLB drivers showed poorer performance on all indicators of simulated driving including an increased number of collisions in the simulator and poorer composite indicators of overall driving performance. A measure of global cognitive function (i.e., the Mini Mental State Exam) was found to be related to the overall driving performance. In addition, measures of attention (i.e., Useful Field of View, UFOV) and space processing (Visual Object and Space Perception, VOSP, Test) correlated significantly with a rater's assessment of driving performance.

## 1. Introduction

Driving an automobile is a primary means of mobility for many older adults, allowing them to access medical services and social contacts and participate in their communities. Driving is associated with independence, quality of life, and better health and, for many older adults, forms a central part of their identity [1]. Research shows that, in Canada, older adults use personal vehicles for almost 90 percent of their daily travel, serving as the driver in approximately 75 per cent of those trips [2]. Driving is so important in the lives of older adults that it is often referred to as an Instrumental Activity of Daily Living (IADL). While it forms a central part of identity and contributes to quality of life, driving among older adults comes with an increased risk of motor vehicle collisions, injury, and mortality due to those collisions [3]. The increased injury risk among older drivers represents a significant public health issue and an economic burden which is particularly troublesome given that, in Canada, drivers over 65 years are the fastest growing segment of the licensed population [4].

Driving is a multifactorial behavior drawing upon multiple physical, cognitive, and sensory domains at the level of the driver [5]. A large and significant body of research points to a relationship between deficits in attention and fitness to drive [6–8]. Age-associated changes in cognition, sensory function, and health increase older drivers' risk of collision, and the identification of at-risk drivers is an important component in reducing traffic-related injuries and fatalities as well as in identifying remediation needs. Cognition is an essential function for safe driving and changes in cognition due to the onset of neurodegenerative conditions can increase older drivers' risk of collision [9].

Some reports suggest that the risk of motor vehicle collisions (MVC) among individuals with disorders that impair cognition has been shown to be comparative to the risk of driving a vehicle under the influence of alcohol. Unlike intoxicated drivers, drivers with cognitive deficits are impaired 24 hours a day, 7 days a week [10–12]. Dementia is the most common cause of cognitive impairment in the older adult population [13]. Dementia is not a normal part of aging; however, estimates suggest that 480,600 Canadians are impacted by dementia (i.e., 1.5% of the Canadian population), a prevalence that is projected to increase to 1,125,200 individuals (i.e., 2.8% of the Canadian population) by 2038 [14]. Drivers with dementia are not a homogenous group as they often start off with mild cognitive impairments and eventually experience more serious declines [13]. Research has demonstrated that, on average, drivers with dementia continue to drive for approximately 4 years after the onset of their symptoms [15]. In addition, it has also been found that one in four Canadians with serious cognitive impairments continue to have a valid driver's license and continue to drive regularly [16].

With the current demographic trends demonstrating an aging population and an associated projected increase in the prevalence of dementia, it is imperative to understand the driving abilities of individuals diagnosed with dementia. Alzheimer's disease (AD) is the most common form of dementia, accounting 70% of the cases [17]. AD is characterized by impairments in reasoning, planning, language, attention, and memory [18]. The body of evidence examining driving performance among individuals with AD has grown in recent years (e.g., [9, 11, 19–21]). For example, our previous work examined simulated driving performance of drivers with mild Alzheimer's disease in comparison to neurologically normal drivers [22]. In particular, we found that mild AD drivers performed poorer in a portable driving simulator in comparison to controls. We also observed that, within the sample of drivers with dementia, measures of attention and visual space perception predicted driving performance better than a test of general cognition (i.e., the Mini-Mental State Exam).

While there is a growing body of literature on the driving abilities of older adults diagnosed with AD, the driving abilities of individuals diagnosed with other types of dementia are poorly understood. Another prevalent form of dementia is Dementia with Lewy Bodies (DLB), accounting for 2–10% of all dementia cases [17, 23, 24]. DLB is a neurodegenerative disease producing a combination of cognitive impairment, parkinsonism, and mental disturbances in the form of hallucinations [25]. The average age of onset of DLB is 67 years, with males being more affected than females [26]. The average duration of the illness is nine years, although estimates of speed and progression vary considerably from study to study [27]. The pathological hallmark of DLB is the presence of Lewy bodies in neurons. In most dementias, such as AD and VaD, memory impairment is the presenting issue. However, in DLB, the most common presenting feature, in 33–65% of all cases, are visual hallucinations [28].

As with most dementias, DLB is marked by a progressive cognitive decline that interferes with normal life. It is postulated that the pattern of dementia is a mixed corticosubcortical dementia since patients have significant frontal-subcortical dysfunction (i.e., impairment of attention, visuospatial function, executive functions, thought, regulatory changes in praxis, and gnosis). Although memory impairments are not usually found in the beginning stages of DLB, they become marked once the disease has fully developed. One of the most impaired cognitive abilities present in DLB is visuoperceptual and spatial functions. Within this domain, DLB patients have particular difficulty with the perception of objects and pictures. It is theorized that this impairment may cause their visual hallucinations, which is a hallmark of the disease [29]. In addition, patients with DLB have difficulty with visual construction and spatial functions, which can be seen through difficulty putting things together (i.e., blocks) or navigating in an environment. In addition to visuoperceptual and spatial impairments, patients with DLB also suffer from attentional deficits. Ballard and colleagues [30] demonstrated that patients with DLB had slowed processing speed, attentional impairments, and fluctuations in attentional impairments.

Given that DLB accounts for approximately 10% of all dementia diagnoses, it is critically important to better understand the driving behaviour of this clinical population as a first step in promoting road safety and reducing the occurrence of costly collisions. Interestingly, Carr and O'Neill [9] note that there are currently no studies that have examined driving behaviour among individuals with DLB. Thus, the purpose of the present study was to examine the simulated driving performance of individuals diagnosed with DLB. It was hypothesized in addition to exhibiting poorer performance in the driving simulator that DLB drivers' deficits in visuospatial skills would emerge as a significant predictor of simulated driving performance.

## 2. Materials and Methods

### 2.1. Participants

A group of individuals diagnosed with mild Dementia with Lewy Bodies (DLB) (*N* = 15) and a group of neurologically healthy older adult controls (*N* = 21) participated in the present study. All participants were over the age of 65 years, English speaking, and held a valid driver's license. The mean age of the mild DLB group was 76.4 years (SD = 6.59) with a range of 68 to 88 years, the mean years of education was 14.20 (SD = 4.55), and the group was comprised of 6 women and 9 men. The mean age of the control group was 77 years (SD = 5.86) with a range of 68 to 86 years, the mean years of education was 13.14 (SD = 3.18), and the group was comprised of 10 women and 11 men. No statistically significant differences were observed between groups in terms of demographic characteristics.

The participants diagnosed with mild DLB were recruited from the Memory Disorders Clinic at the Bruyère Continuing Care Center (i.e., a tertiary care facility) in Ottawa, ON (Canada). The patients were assessed for severity using the Global Deterioration Rating Scale and only participants in the mild stage of DLB were included in this study (i.e., ≤stage 3). Patients taking psychoactive medications, such as acetylcholinesterase inhibitors, were included in the DLB group. The exclusion criteria included serious visual or hearing impairments left uncorrected (e.g., cataracts), serious health problems aside from dementia (e.g., mental illnesses, history of head injury, epilepsy, apoplexy, heart attacks, hypertension, and sleep apnea), any history of substance abuse, and any history of learning disabilities.

The convenience sample of neurologically healthy older drivers (control group) was recruited through announcements placed in a local community newspaper. Control participants taking any medications that could alter cognitive abilities were excluded. None of the control participants had abnormal Mini-Mental State Exam (MMSE) scores of less than 25.

### 2.2. Measures

#### 2.2.1. Global Functioning

*Mini Mental State Exam (MMSE) [31]*. The MMSE is one of the most widely used brief screening instruments in dementia.

*Mattis Dementia Rating Scale (DRS-2 and DRS-2-Alternate) [32].* The DRS-2 assesses attention, memory, visuospatial construction, conceptualization, and initiation/perseveration. The test was administered according to the discontinue rules as outlined in the test protocol.

#### 2.2.2. Visuospatial/Perceptual Abilities

*Visual Object and Space Perception (VOSP) Test [33].* This is a measure of visuoperceptual and spatial abilities that specifically assesses object and space perception. All eight subtests of this battery were administered. A VOSP object perception composite score was calculated by adding the first four subtests and a VOSP space perception composite score was calculated by adding the last four subtests. These calculations were extracted from the user manual and were found to be valid and reliable measures of object perception and space perception [34].

#### 2.2.3. Tests of Attention

*Test of Everyday Attention (TEA) [35].* Evidence has shown that the factor structure of the TEA aligns with contemporary research supporting several attentional circuits in the brain [36]. These factors are* sustained attention*,* selective attention*, and* attentional switching*.

The scores from the eight subtests in the TEA were aggregated so that they matched the three factors outlined in Robertson et al. [36]. Standard scores (i.e., *Z*-scores) were first computed for each subtest and, subsequently, variables corresponding to each of the three factors were summed.

*Useful Field of View (UFOV) [37]*. The UFOV© task is a computerized measure of attention that examines three test variables and is composed of three subtests, entitled processing speed, divided attention, and selective attention. Each subtest presents participants with stimuli for increasingly shorter durations and, after each presentation, participants are asked to indicate what they had seen. Performance on the UFOV is often cited as being related to performance on functional activities requiring sustained attention, such as driving [38].

#### 2.2.4. Simulated Driving

Participants were asked to complete a simulated driving scenario mimicking an on-road evaluation. The STISIM Drive software (Version 2.08.004; Systems Tech, California) was implemented on a Dell Precision M6300 Laptop Computer (Intel Core 2 DUO Processor, 2.10 GHz/2.0 GB RAM) with a 17-inch display running Windows XP. The laptop was equipped with a Logitech brake/throttle and steering wheel (model G25). Instructions to the drivers (e.g., turn left/right, lane changes, and speed maintenance, etc.) were given through laptop speakers. The driving simulator had a 60-degree horizontal field of view and a 75-degree vertical field of view and the frame rate of the simulator was 30 frames per second (30 Hz) (see [22] for more information).

Before beginning, participants completed a comprehensive training session including a thorough explanation of the task, an accommodation phase during which they practiced operating the pedals and steering wheel, followed by a training course that took approximately 20 minutes to complete (see [39] for a detailed description).

The assessment course employed was programmed to mimic a driving assessment by a provincial regulatory body in Canada and is described by Weaver et al. [8]. It was 12.3 kilometers long, based on a real segment of road found in Thunder Bay, Ontario, and included driving in residential, highway, and urban environments. This particular assessment course was selected in our study for several reasons. Notably, given that the course is utilized by a provincial licensing body to differentiate between safe and unsafe drivers, it was found to exhibit high face validity. Similarly, the evaluation course allowed for the collection of two aggregate measures of driving performance: the total number of errors recorded by the simulator and a structured rater score. The course presented participants with a wide variety of typical driving situations allowing for generalizability. Most importantly, previous research examining driving behavior among drivers from across the driving lifespan (ages 18–83 years) shows that scores on the simulated assessment course correlate highly with those on the real-world driving course [40].

Measurements of the simulated driving task were generated both from driving performance as recorded by the computer and from a demerit point assessment. Driving errors and parameters were recorded (e.g., speed exceedances, stops sign violations, and traffic light violations) by the simulator. The driving related variables collected by the driving simulator are presented in Table 1.

All simulated drives were recorded and scored by two blind independent raters using a structured demerit point assessment. The two blinded raters independently assessed a video playback of the simulated drive. Interrater reliability was found to exceed *r* = .9 and as such the mean of both scores was used in the analysis.

None of the participants in this study reported any symptoms associated with using the driving simulator, a condition known as simulator adaptation syndrome (SAS).

### 2.3. Procedure

Patients at the tertiary care facility with a diagnosis of mild DLB were contacted in order to verify whether they were willing to participate in the present study and verify that they met the inclusion criteria. Following this screening, testing commenced immediately.

Testing sessions lasted approximately two and a half hours. All participants underwent cognitive and computerized assessment including a test of general cognitive functioning (DRS-2), visuospatial/perceptual abilities (VOSP), attention (TEA and UFOV), and processing speed (UFOV). Participants also completed a simulated driving assessment. All cognitive and computerized testing was completed according to the protocol specified by each test. The cognitive and computerized testing was administered in the presence of the participant and the investigator only.

Following data collection, each drive was rated independently by two raters using the playback feature of the STISIM Drive.

## 3. Results

### 3.1. Driving Performance

The two groups of participants were compared using a series of between groups ANOVAs with group (DLB and control group) as the primary independent variable.

Comparison of the driving variables (Table 1) revealed that DLB drivers consistently performed poorer in comparison to neurologically healthy older drivers. The composite measures of driving performance offer a global indication of participants' driving behaviour in the context of the simulated assessment course. The results revealed that the number of errors accrued by the driving simulator was significantly higher among DLB drivers (*M* = 57.07) than among controls (*M* = 27.86) [*F*(1,34) = 57.61,  *p* < .001]. Similarly, DLB drivers were assigned over four times the number of demerit points (*M* = 284.00) in comparison to the control group (*M* = 59.64) during the simulated driving assessment, a difference that also reached statistical significance [*F*(1,36) = 57.61, *p* < .001].

To better understand the composite results, types of driving errors were compared between groups. The results showed that DLB drivers exceeded the posted speed limit significantly more often in comparison to controls [*F*(1,34) = 7.53, *p* = .01] and spent an average of 24% of the time driving over the speed limit, a significantly higher proportion than controls who spent an average of 12% of the time over the speed limit [*F*(1,34) = 6.82, *p* = .01]. In terms of lateral control of the vehicle, DLB drivers crossed the centerline [*F*(1,34) = 30.39, *p* < .001] more frequently than controls and were found to spend an average of 13% out of their own lane, significantly higher than controls who spent an average of 3% out of their own lane [*F*(1,34) = 9.01, *p* = .01]. DLB drivers exceeded the road edge more often than did neurologically healthy drivers [*F*(1,34) = 9.17, *p* = .01].

Comparison of performance between DLB and controls revealed that DLB drivers failed to stop at traffic lights significantly more often than controls [*F*(1,34) = 2.47, *p* < .001]. DLB drivers were also found to have difficulty in interacting safely with other road users in the driving simulator as evidenced by significantly more crashes compared to controls [*F*(1,34) = 19.83, *p* < .001].

### 3.2. Cognitive Measures

Prior to calculating the degree of association between the cognitive measures with indicators of driving performance, the cognitive measures were first compared between the two groups. To this end, ANOVAs were again computed for all cognitive measures with group (DLB and control group) as the primary factor.

In comparison to controls, DLB drivers were found to have poorer performance on all measures of cognitive function administered in this study. The descriptive statistics for each measure and test group are presented in Table 2.

### 3.3. Association between Cognitive Function and Simulated Driving among DLB Drivers

One goal of this study was to assess the contribution of cognitive tests of global cognition, attention, and space processing in assessing driving outcomes among individuals with DLB. To meet this goal,* Pearson correlation coefficients* were calculated between each of the cognitive test scores and key indicators of overall driving performance for the DLB group only.

Table 3 summarizes the correlations between cognitive tests and driving outcomes among individuals with DLB. In terms of global measures of cognitive function, the results indicated statistically significant relationships between the MMSE and the total number of errors in the simulator (*r* = −.529, *p* = .043) and the rater score (*r* = −.622, *p* = .013). When associations between the MMSE and types of errors were examined, it is clear that this relationship derives primarily from its association with the percentage of time spent over the speed limit (*r* = −.656, *p* = .008) and the percentage spent out of the lane (*r* = −.792, *p* < .001). In particular, DLB drivers with lower MMSE scores were associated with more driving time over the speed limit and more time spent outside of the driver's lane.

The DRS, however, was not significantly correlated with simulated driving outcomes among DLB participants. In terms of visual processing, a statistically significant correlation was observed between the VOSP space scores and the rater score (*r* = −.624,  *p* = .013). The associations between measures of attention and simulated driving indicated a statistically significant association between the first subtest of the UFOV (i.e., processing speed) and the rater score (*r* = .548, *p* = .034). The third subtest of the UFOV (i.e., selective attention) was also significantly related to the number of speed exceedances in the driving simulator (*r* = .588, *p* = .021).

## 4. Discussion

The purpose of the current study was to examine the driving behaviour among older drivers with mild DLB in comparison to neurologically healthy older drivers. To our knowledge, this is the first empirical study to examine the driving behaviour of drivers diagnosed with DLB. Our results indicated clearly that when variables related to simulated driving performance were contrasted between groups, DLB drivers were found to perform significantly worse on all measures. Thus, the simulated driving performance of mild DLB drivers was found to be significantly impaired, which is a noteworthy result considering that most drivers diagnosed with dementia continue to drive for 4 years following diagnosis [15].

When performance on the cognitive tests was contrasted between groups, drivers with mild DLB were found to be impaired across all measures that were administered. This finding aligns with the existing literature indicating that attention, visuospatial and perceptual skills, and global cognition are impacted within this patient group [25]. However, what is most interesting in our results is the degree of association between scores on cognitive tests and simulated driving performance. In particular, a strong association was found between the MMSE and the total number of errors in the simulator, as well as with the rater score. When examining MMSE scores in relation to specific errors in the simulator, it was found that mild DLB drivers with lower MMSE scores were associated with driving over the speed limit and outside of the driver's lane. Visuospatial processing was also found to be associated with rater score. In addition, measures of attention, including processing speed and selective attention, significantly correlated with rater score and speed exceedances, respectively.

These results are of particular relevance in that they demonstrate that impaired cognitive abilities, namely, global cognition, attention, and visuospatial functioning, are predictive of poor simulated driving performance among mild DLB drivers. These findings support the existing literature examining driving performance of individuals with dementia, showing that, in comparison to neurologically healthy controls, AD drivers perform poorer in the driving simulator (for a detailed summary of the existing literature, see [21]). Indeed, the authors of this study recently completed a similar investigation that examined the driving performance of drivers with mild AD, using an identical driving simulator protocol as the one described herein. In particular, Yamin, Stinchcombe, and Gagnon (accepted with revision) found that drivers with mild AD exhibited poorer performance in the simulator on all measures except for the number of road edge excursions. Global cognition scores (i.e., MMSE and DRS), attention tests and visual perception results of AD drivers were also found to be lower than what was observed in the group of healthy controls. In contrast, AD drivers did not display deficits of space perception. Moreover, among drivers with mild AD, strong associations were found with most measures of attention and a measure of visual perception but not with measures of global cognition. Alongside our colleagues in the field of road safety, we cautioned against the use of measures of global cognition in isolation to identify unsafe drivers with dementia given our previous results with a sample of mild AD drivers [41, 42]. However, in the present study, one of the stronger associations with driving performance that we found was with a measure of global cognition (i.e., MMSE). It is relevant to note that mild AD and mild DLB samples showed similar levels of decline as measured by the Global Deterioration Scale (GDS) (i.e., a score of 3 on the GDS indicating mild dementia). Considering this result as well as the pattern of findings that emerged when examining simulated driving performance among drivers with mild AD, these data suggest that drivers with mild DLB exhibit some global cognitive decline that impacts driving performance. As indicated in Introduction, driving is a multifactorial behaviour drawing upon multiple cognitive, sensory, and physical systems [5]. It follows that when patients experience global decline, driving performance will be negatively impacted. Within the sample of mild DLB drivers, we observed that global cognition accounts for a substantial proportion of the variance in simulated driving performance. While we do not recommend that the MMSE be used in isolation to predict driving performance, our results highlight the utility of this measure among mild DLB drivers.

A limitation of this study is that no on-road assessment was completed. However, this study employed a portable driving simulator, offering several advantages. First, driving in a simulator is less stressful than an on-road assessment for participants. Second, a small portable simulator reduces the risk of negative symptoms associated with using the driving simulator (i.e., simulator adaptation syndrome, SAS). Third, driving simulators are considered to be a safe and economical means of presenting drivers with consistent situations and collecting behavioural data in real time. Finally, simulator performance has been shown to be reliable and a valid indication of on-road performance [40]. Importantly, we used a scenario that was developed to assess the various components routinely assess in an on-road driving examination. Nevertheless, in this study, we did not collect on-road data and thus the generalizability of our findings to real-world driving and collision risk should be extrapolated with caution. It would be of interest for future research to examine on-road driving performance among mild DLB drivers. Such an investigation would enhance the clinical relevance of our findings in order to inform policy and practice related to fitness to drive.

## 5. Conclusions

This study was the first to shed light on the driving behaviour of individuals diagnosed with mild DLB. The results highlight the importance of cognitive functions, notably, global cognition, spatial and attentional abilities, in predicting driving ability. While a diagnosis of dementia does not automatically imply the immediate removal of driving privileges, it may alert professionals to the need for further in-depth assessment and regular monitoring. Based on these data, global cognition, attention, and spatial abilities appear to be most related to driving performance among this clinical population. Future research directions should examine the clinical relevance of these findings with respect to on-road performance and crash risk.

## Figures and Tables

**Table 1:** Differences in simulated driving performance between participants with Dementia with Lewy Bodies (DLB) and controls.

Variable classification	Variable	DLB group		Control group		df	*F*	*p*
		Mean DLB	SD DLB	Mean Ctrls	SD Ctrls			
Intersection behaviour	Total number of traffic light tickets	2.47	.52	0.43	0.60	1, 34	113.62	<.001

Speed	Number of speed exceedances	17.00	5.64	12.24	4.74	1, 34	7.53	.01
	Over the speed limit percent of time	23.78	16.53	12.95	8.04	1, 34	6.82	.01

Lateral control	Total number of centerline crossings	9.67	4.34	2.62	3.34	1, 34	30.39	<.001
	Total number of road edge excursions	17.80	10.43	7.52	9.76	1, 34	9.17	.01
	Out of lane percent of time	12.65	12.59	3.46	5.40	1, 34	9.01	.01

Crash	Total number of crashes	3.27	1.75	1.05	1.24	1, 34	19.83	<.001

Composite indicators	Rater score	284.17	80.00	107.74	59.64	1, 34	57.61	<.001
	Simulator errors	57.07	18.03	27.86	16.18	1, 34	25.95	<.001

**Table 2:** Differences in cognitive performance between participants with Dementia with Lewy Bodies (DLB) and controls.

Variable classification	Variable	DLB group		Control group		df	*F*	*p*
		Mean DLB	SD DLB	Mean Ctrls	SD Ctrls			
Global functioning	Mini-Mental State Exam (MMSE)	22.40	2.80	29.00	1.30	1, 34	90.24	<.001
	Dementia Rating Scale (DRS)	108.60	17.57	136.38	4.43	1, 34	48.72	<.001

Visual processing	Visual Object and Space Perception (VOSP) Test, Object	61.20	4.35	67.95	5.82	1, 34	14.41	.01
	Visual Object and Space Perception (VOSP) Test, space	35.20	10.58	45.48	4.62	1, 34	15.76	<.001

Attention	Test of Everyday Attention (TEA), visual selection	−2.22	.59	.97	1.90	1, 34	58.61	<.001
	Test of Everyday Attention (TEA), sustained attention	−2.02	2.41	1.14	1.16	1, 34	39.52	<.001
	Test of Everyday Attention (TEA), switching attention	−2.25	2.40	1.94	1.81	1, 34	29.92	<.001
	Useful Field of View (UFOV), processing speed	272.07	120.32	24.91	16.74	1, 34	87.25	<.001
	Useful Field of View (UFOV), divided attention	432.20	135.17	152.52	116.45	1, 34	44.16	<.001
	Useful Field of View (UFOV), selective attention	490.20	37.96	304.76	115.13	1, 34	35.86	<.001

**Table 3:** Pearson correlation coefficients indicating relationship between tests of cognitive function and simulated driving outcomes for the DLB group.

Variable	Rater score *r* (*p*)	Errors in the simulator *r* (*p*)	Number of crashes *r* (*p*)	Total number of traffic light tickets *r* (*p*)	Number of speed exceedances *r* (*p*)	Over the speed limit percent of time *r* (*p*)	Total number of centerline crossings *r* (*p*)	Total number of road edge excursions *r* (*p*)	Out of lane percent of time *r* (*p*)
Mini-Mental State Exam (MMSE)	−.622 (.013)	−.529 (.043)	−.329 (.230)	−.484 (.067)	.095 (.736)	−.656 (.008)	−.430 (.110)	−.487 (.066)	−.792 (<.001)

Dementia Rating Scale (DRS)	−.445 (.096)	.055 (.846)	−.347 (.205)	−.309 (.263)	.092 (.744)	−.107 (.704)	−.300 (.277)	.182 (.516)	−.159 (.571)

Visual Object and Space Perception (VOSP) Test, object	−.050 (.859)	−.162 (.563)	−.280 (.313)	−.076 (.787)	.047 (.869)	−.030 (.915)	−.227 (.415)	.037 (.895)	−.267 (.336)

Visual Object and Space Perception (VOSP) Test, space	−.624 (.013)	−.113 (.689)	−.385 (.157)	−.332 (.226)	.000 (1.000)	−.192 (.493)	−.310 (.261)	.048 (.864)	−.097 (.731)

Test of Everyday Attention (TEA), visual selection	−.308 (.264)	−.325 (.238)	−.040 (.888)	−.125 (.658)	−.074 (.793)	−.319 (.246)	−.067 (.813)	−.537 (.039)	−.315 (.253)

Test of Everyday Attention (TEA), sustained attention	−.192 (.493)	.059 (.835)	−.177 (.528)	−.111 (.694)	−.109 (.700)	.255 (.358)	−.078 (.783)	.276 (.319)	.148 (.598)

Test of Everyday attention (TEA), switching attention	−.096 (.732)	.144 (.609)	.029 (.918)	−.017 (.952)	−.027 (.923)	−.301 (.276)	−.028 (.922)	.339 (.217)	.039 (.890)

Useful Field of View (UFOV), processing speed	.548 (.034)	.427 (.113)	−.453 (.090)	.280 (.312)	.344 (.210)	.256 (.357)	.321 (.243)	.221 (.428)	.256 (.357)

Useful Field of View (UFOV), divided attention	.510 (.052)	.463 (.082)	.207 (.460)	.301 (.275)	.401 (.138)	.410 (.129)	.242 (.386)	.338 (.218)	.410 (.129)

Useful Field of View (UFOV), selective attention	.481 (.069)	.507 (.053)	.358 (.190)	.250 (.369)	.588 (.021)	.240 (.389)	.361 (.186)	.287 (.301)	.240 (.389)
